# A Comparison of Univariate and Multivariate Forecasting Models Predicting Emergency Department Patient Arrivals during the COVID-19 Pandemic

**DOI:** 10.3390/healthcare10061120

**Published:** 2022-06-16

**Authors:** Egbe-Etu Etu, Leslie Monplaisir, Sara Masoud, Suzan Arslanturk, Joshua Emakhu, Imokhai Tenebe, Joseph B. Miller, Tom Hagerman, Daniel Jourdan, Seth Krupp

**Affiliations:** 1 Department of Marketing & Business Analytics, San Jose State University, One Washington Square, San Jose, CA 95192, USA; 2 Department of Industrial & Systems Engineering, Wayne State University, 4815 4th Street, Detroit, MI 48202, USA; leslie.monplaisir@wayne.edu (L.M.); saramasoud@wayne.edu (S.M.); jemakhu@wayne.edu (J.E.); 3 Department of Computer Science, Wayne State University, 5057 Woodward Ave., Detroit, MI 48202, USA; suzan.arslanturk@wayne.edu; 4 Texas Commission on Environmental Quality, Critical Infrastructure Division, 1200 Park 35 Circle, Austin, TX 78711, USA; yoshearer@gmail.com; 5 Departments of Emergency Medicine and Internal Medicine, Henry Ford Hospital, 2799 W Grand Blvd, Detroit, MI 48202, USA; jmiller6@hfhs.org (J.B.M.); thagerm1@hfhs.org (T.H.); djourdan2@hfhs.org (D.J.); skrupp1@hfhs.org (S.K.)

**Keywords:** COVID-19, emergency department, forecasting, deep learning, emerging infectious disease

## Abstract

The COVID-19 pandemic has heightened the existing concern about the uncertainty surrounding patient arrival and the overutilization of resources in emergency departments (EDs). The prediction of variations in patient arrivals is vital for managing limited healthcare resources and facilitating data-driven resource planning. The objective of this study was to forecast ED patient arrivals during a pandemic over different time horizons. A secondary objective was to compare the performance of different forecasting models in predicting ED patient arrivals. We included all ED patient encounters at an urban teaching hospital between January 2019 and December 2020. We divided the data into training and testing datasets and applied univariate and multivariable forecasting models to predict daily ED visits. The influence of COVID-19 lockdown and climatic factors were included in the multivariable models. The model evaluation consisted of the root mean square error (RMSE) and mean absolute error (MAE) over different forecasting horizons. Our exploratory analysis illustrated that monthly and weekly patterns impact daily demand for care. The Holt–Winters approach outperformed all other univariate and multivariable forecasting models for short-term predictions, while the Long Short-Term Memory approach performed best in extended predictions. The developed forecasting models are able to accurately predict ED patient arrivals and peaks during a surge when tested on two years of data from a high-volume urban ED. These short- and long-term prediction models can potentially enhance ED and hospital resource planning.

## 1. Introduction

Emergency departments (ED) are susceptible to significant variations in patient arrival times. At times, EDs experience “surges” of a large influx of patients. Surges are usually the result of human-made or natural events, such as the COVID-19 pandemic. The strain of unexpected and substantial fluctuations in patient volume can cause long patient waiting times [1] and long boarding times while awaiting placement in the hospital [2]. ED crowding results from a mismatch between existing hospital capacity and various input, throughput, and output factors, such as ED arrivals, beds, staffing, hospital admission, and discharge rates. Inadequate handling of patient crowding may lead to suboptimal ED operations linked to adverse patient outcomes, such as new or worsening symptoms or death [3,4]. Additionally, ED crowding causes strain on medical staff and is associated with reduced patient safety [1,5,6].

The COVID-19 pandemic has intensified existing ED crowding and throughput issues. Many hospitals and EDs have faced unprecedented challenges in managing surges in infected patients, leaving them overwhelmed and unable to meet patient care demands promptly. This challenge has been exacerbated by the nursing shortage. To maintain high-quality care and manage the increase in ED volumes during surges, as seen during the COVID-19 pandemic, hospitals and EDs could benefit from high-quality forecasting data.

ED demand prediction, expressed as daily visits, has been assessed using different time-series forecasting approaches [7,8]. Although such studies exist on forecasting ED arrivals before the pandemic [9], much of the variation in ED arrivals remains unaccounted for, and model derivation during surges in patient encounters is lacking [10]. Furthermore, it is well established in literature that climatic variables, such as temperature, can affect the health of a community, leading to an increase in ED patient arrivals and hospital admissions [11,12,13,14]. There is a need to understand if climatic variables significantly affect patient arrivals during the pandemic.

The primary objective of this study was to forecast ED arrivals during a pandemic over different time horizons. The secondary objective was to compare the performance of the four forecasting models in predicting the demand for medical care, considering the effect of a global pandemic and climatic factors on patient arrival to the ED. Such forecasting and comparative modeling have the potential to advance the science of predicting ED and hospital resource utilization before surges in patient encounters.

## 2. Materials and Methods

### 2.1. Study Design, Setting, and Selection of Participants

We derived forecasting models for the daily number of ED visits in a retrospective, observational, cross-sectional study. These models compared different time horizons ranging from 1 to 30 days to predict daily patient arrivals. For example, a 14-day horizon predicted patient arrivals in 14 days from the time the model was run. We compared the model performance with the inclusion of pandemic and climatic factors. Data collection was inclusive of encounters from January 2019 to December 2020. The study was approved by the Henry Ford Hospital Institutional Review Board prior to data collection, with a waiver of informed consent.

The data were collected from an 877-bed urban academic hospital with a Level 1 trauma center serving a high-acuity, diverse, urban patient population in Detroit, Michigan. The ED treats an estimated 100,000 patients annually and has a 24% admission rate. All patients were included during the study period. We did not exclude any patients.

**Figure 1:**
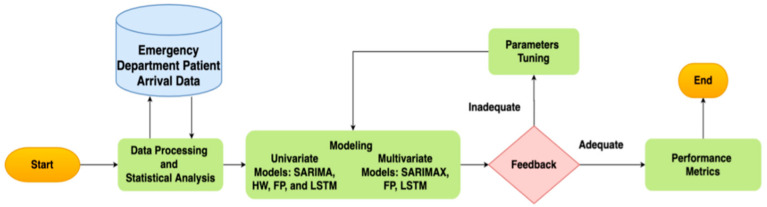
Forecasting modeling framework for ED patient arrivals. The proposed method has three main parts: data processing/statistical analysis, model building, and evaluation.

Although this study builds on existing forecasting methods, our use-inspired analytics research aims to apply these methods to solve a unique and challenging healthcare operation problem during the ongoing COVID-19 pandemic. This work presents a retrospective study, applying predictive models to accurately forecast patient arrival, as portrayed in Figure 1.

### 2.2. Data Processing and Statistical Analysis

As shown in Figure 1, the framework starts with data processing and statistical analysis. The second phase focused on the development of predictive models. The predictive models included univariate models: seasonal autoregressive integrated moving average (SARIMA), Facebook Prophet (FP), Holt–Winters (HW), and Long Short-Term Memory (LSTM). They also included multivariable models: seasonal autoregressive integrated moving average exogenous (SARIMAX), FP with regressors, and exogenous LSTM. The forecasting algorithms were evaluated based on their performance on the test dataset using the root mean square error (RMSE) and mean absolute error (MAE).

From the de-identified data, we extracted the arrival time and date for all patients arriving at the ED. To investigate the variation in patients’ arrival, including the impact by month of the year and day of the week, we used analysis of variance (ANOVA). As part of the exploratory analysis, we applied a Bayesian change-point analysis to investigate the behavior of patients’ arrival to the ED. Next, we analyzed the time-series data for stationarity. As stationarity has a tremendous influence on how the data are perceived and predicted, first-order differencing was applied to stabilize the time-series mean and/or variance if the time-series data are non-stationary. We used the Augmented Dickey Fuller (ADF) test to analyze the stationarity of the transformed data. Finally, we used a Spearman correlation to assess the relationship between the time-series variables for feature selection analysis in multivariable forecasting.

### 2.3. Forecasting Models

Time-series forecasting is a machine learning strategy in which models are trained over time-sequenced data (i.e., time-series) to make predictions [15]. Time-series forecasting has many applications, such as disease prevention and incidence [16], finance (i.e., predicting future stock or sale prices) [17], weather forecasting (i.e., monitoring air pollution) [18], and transportation (i.e., predicting traffic flow) [19]. To develop forecasting models for time-series analysis, the ED patient arrival time-series data (T) can be modeled as a matrix, where $T=\left[\left[t_{1}],[t_{2}],[t_{3}],\ldots ,[t_{n}\right]\right]$ and each element, $T_{n}$, is a vector.

Patient arrival can be studied using either univariate or multivariable time-series forecasting. In the univariate time-series analysis, single observations of patient arrival were recorded sequentially over daily increments, and the forecasting model contained lag values of daily patient arrival as independent variables. Multivariable time-series models are extensions of the univariate case, incorporating the lags of other time series in addition to patient arrival at the same time increments. Table 1 presents the benefits and limitations of the models.

Multivariable time-series forecasting models study the interrelationships among time-series variables. Table 2 reports the exogenous variables implemented in the multivariable models. The influence of an emerging infectious disease outbreak and climatic features were the main factors included in the multivariable model. This study extracted historical climatic data from the National Weather Service Archive [20]. Literature supports the impact of climatic variables, such as temperature, on ED daily visits, which correlates with demand for medical services [21,22]. In addition to the climate factors, data on the COVID-19 pandemic are relevant to ED visits [23]. The timeframe of the disease outbreak and subsequent lockdown in Michigan was extracted from the Michigan Department of Human and Health Services [24].

A brief summary for the univariate and multivariable forecasting models are presented below. See the methodological equations section of Appendix A for a detailed explanation of the forecasting models.

Seasonal Autoregressive Integrated Moving Average (SARIMA). The SARIMA model is an extension of ARIMA which accounts for seasonality in time series data [25]. SARIMA captures patients’ arrival behavior based on historical time-series data and is widely applied in healthcare-related forecasting [26]. SARIMA is synonymous with a simple linear regression model and only accounts for one independent variable. The time series function, *Y_t_*, utilizes a lag operator, *B*, to process SARIMA as (p,d,q)×(P,D,Q)m. The SARIMA model equation is [25]:

(1) $$\emptyset_{p}\left(B\right)\Phi_{P}{\left(B^{m}\right)}^{d}{\left(1-B^{m}\right)}^{D}Y_{t}=\theta_{q}\left(B\right)\Theta_{Q}\left(B^{m}\right)\varepsilon_{t}$$

In Equation (1), B is the lag operator (defined as $B^{k}\times Y_{t}=Y_{t-k}$)

(2) $$\emptyset_{p}\left(B\right)=1-\emptyset_{1}B-\emptyset_{2}B^{2}-\ldots -\emptyset_{p}B^{p}$$

(3) $$\Phi_{P}\left(B^{m}\right)=1-\Phi_{m}B^{m}-\Phi_{2m}B^{2m}-\ldots -\Phi_{Pm}B^{Pm}$$

(4) $$\theta_{q}\left(B\right)=1-\theta_{1}B-\theta_{2}B^{2}-\ldots -\theta_{q}B^{q}$$

(5) $$\Theta_{Q}\left(B^{m}\right)=1-\Theta_{m}B^{m}-\Theta_{2m}B^{2m}-\ldots -\Theta_{Qm}B^{Qm}$$

where ϕ(B) and θ(B) are polynomials of order *p* and *q*, respectively. $\Phi \left(B^{m}\right)\;\mathrm{and}\;\Theta \left(B^{m}\right)$ are polynomial in B of degrees P and Q, respectively. p denotes the order of non-seasonal autoregression, d is the number of regular differences, and q is the order of the non-seasonal moving average. P means the order of seasonal autoregression, D is the number of seasonal differences, Q represents the order of the seasonal moving average, and m denotes the length of the season.

Seasonal Autoregressive Integrated Moving Average Exogenous (SARIMAX). The SARIMAX model expands the capabilities of SARIMA to cover the interrelations of exogenous variables (i.e., more than one independent variable) [27,28]. SARIMAX models consider exogenous factors in search of a better justification of the behavior of the target variable (i.e., patients’ arrival). It provides the required modeling framework to rectify autocorrelation by describing error terms of linear regression models, expressed as (p, d, q)×(P, D, Q)m. SARIMAX has the potential to be a good fit for modeling ED patient arrivals as they exhibit a seasonal pattern, and the effect of COVID-19 and climatic factors can be modeled as an exogenous variable that affects daily ED visits. The SARIMAX is modeled as:

(6) $$Y_{t}=\beta_{0}+\beta_{1}X_{1,t}+\beta_{2}X_{2,t}+\ldots +\beta_{k}X_{k,t}+\frac{\left(1-\theta_{1}B-\theta_{2}B^{2}-\ldots -\theta_{q}B^{q}\right)\left(1-\Theta_{1}B^{s}-\Theta_{2}B^{2s}-\ldots -\Theta_{Q}B^{Qs}\right)}{\left(1-\phi_{1}B-\phi_{2}B^{2}-\ldots -\phi_{p}B^{p}\right)\left(1-\Phi_{1}B^{s}-\Phi_{2}B^{2s}-\ldots -\Phi_{P}B^{Ps}\right)}\varepsilon_{t}$$

where $Y_{t}$ is the *t*th observation of the dependent variable; $X_{1,t},\;X_{2,t},\;\ldots ,\;X_{k,t}$ expresses the corresponding observations of the explanatory (exogenous) variables; $\beta_{0},\;\beta_{1},\;\beta_{2},\;.\;.\;.\;,\;\beta_{k}$ denotes parameters of the regression part; and $\varphi_{1},\;\varphi_{2},\;.\;.\;.\;,\;\varphi_{p},\;\Phi_{1},\;\Phi_{2},\;.\;.\;.\;,\;\Phi_{P},\;\theta_{1},\;\theta_{2},\;.\;.\;.\;,\;\theta_{q},\;\mathrm{and}\;\Theta_{1},\;\Theta_{2},\;.\;.\;.\;,\;\Theta_{Q}$ represents the weights for the non-seasonal and seasonal autoregressive terms and moving average terms. SARIMAX seems to be a good fit in the present study, as ED patient arrival exhibits a seasonal pattern, as does the COVID-19 lockdown, and climatic factors can be modeled as an exogenous variable that affects daily ED visits.

Facebook Prophet (FP). FP was developed and introduced by Facebook in 2017. FP is a method for forecasting time series data using an additive model, where nonlinear trends fit daily, weekly, and yearly seasonality, including the effects of events [29]. FP utilizes a generalized linear and additive regression model y(t) comprising the following components:

(7) $$y\left(t\right)=g\left(t\right)+s\left(t\right)+h\left(t\right)+\epsilon_{t}$$

where trend, g(t), is the non-periodic changes; seasonality, s(t), represents the periodic changes; the holiday component, h(t), contributes information about events occurring within the ED patient arrival data and as an extra regressor. The error term, $\epsilon_{t},$ represents any distinctive features of the data that the model does not fit. The FP trend function, *g*(*t*), can be denoted as a piecewise linear growth model or a saturating growth model. Since patient arrival does not exhibit a saturating growth, a piecewise linear growth model is utilized:

(8) $$g\left(t\right)=\left(k+a{\left(t\right)}^{T}\times \delta \right)t+\left(m+a{\left(t\right)}^{T}\times \gamma \right)$$

where k is the growth rate; δ is the rate adjustment; m is an offset parameter; and γ is the trend changepoints, $s_{j}$, and is set as $-s_{j}\delta_{j}$, with a(t) defined as:

(9) $$a_{j}\left(t\right)=\{\begin{array}{c} 1\;if\;t\geq s_{j}\;\\ 0\;otherwise \end{array}$$

The changepoints allow us to adjust the resulting forecast based on experience. Therefore, the trend of the forecast can be fine-tuned, which results in an improved forecast. The seasonality function s(t) can be analyzed and fit into the proposed model with seasonality effects (i.e., daily, weekly, and yearly) using the Fourier series. The seasonality equation is given as:

(10) $$s\left(t\right)=\overset{N}{\underset{n=1}{\sum }}\left(a_{n}\times \cos\left(\frac{2\pi \mathrm{nt}}{P}\right)+b_{n}\times \sin\left(\frac{2\pi \mathrm{nt}}{P}\right)\right)$$

where P is the regular period of 365 days for the yearly seasonality pattern. Additionally, FP allows the inclusion of explanatory variables to enhance the forecast results. In this study, the events are modeled as the COVID-19 pandemic period. For instance, using the h(t) function and defining the dates of the pandemic as a matrix of regressors, Z(t) is defined as:

(11) $$Z\left(t\right)=\left[1\left(t\;\in D_{1}\right),\;\ldots ,\;1\left(t\;\in D_{L}\right)\right]$$

(12) $$h\left(t\right)=Z{\left(t\right)}_{k}$$

where D is the set of pandemic dates, $\kappa \sim Normal(0,\;v^{2}$), and *v* is the event smoothing parameter. For the multivariable FP model, additional variables such as the maximum temperature, average temperature, minimum temperature, pressure, humidity, and precipitation were utilized, resulting in a more reliable forecast.

Holt–Winters (HW). The HW method models the patients’ arrival in three dimensions: a typical value (average), a slope (trend) over time, and seasonality. It encompasses forecast and smoothing equations—one for the level, $\ell_{t}$; one for the trend, $b_{t}$; and one for the seasonal component, $s_{t}$, with corresponding smoothing parameters, $\alpha ,\;\beta^{*},\;\mathrm{and}\;\gamma$. c is used to denote the seasonality frequency (i.e., the number of seasons in a year in which patients present to the ED). Two variations exist for the HW method, namely additive HW and multiplicative HW. The additive HW method is ideal when seasonal variations are constant through the series, whereas the multiplicative HW method is ideal when seasonal variations are changing proportionally to the level of the series [22,30]. In this study, the seasonal multiplicative HW method was used as it exhibited a better fit to the data. The equation for the multiplicative HW form is expressed as [31]:

(13) $${\hat{y}}_{\left(t+h|t\right)}=\left(\ell_{t}+hb_{t}\right)s_{t+h-c\left(k+1\right)}$$

(14) $$\ell_{t}=\alpha \times \frac{y_{t}}{s_{t-c}}+\left(1-\alpha \right)\times \left(\ell_{t-1}+b_{t-1}\right)$$

(15) $$b_{t}=\beta^{*}\times \left(\ell_{t}-\ell_{t-1}\right)+\left(1-\beta^{*}\right)\times b_{t-1}$$

(16) $$s_{t}=\gamma \times \frac{y_{t}}{\left(\ell_{t-1}+b_{t-1}\right)\;}+\left(1-\gamma \right)\times s_{t-c}$$

where 0<α ≤ 1, 0 ≤ β ≤ 1 and 0 ≤ γ ≤ 1–α. The $\ell_{t}$ values represent the baseline, the $b_{t}$ values represent the trend, and the $s_{t}$ values represent the seasonality component. In the multiplicative model, for any consecutive c periods, the sum of $s_{t}\approx 1$.

**Figure 2:**
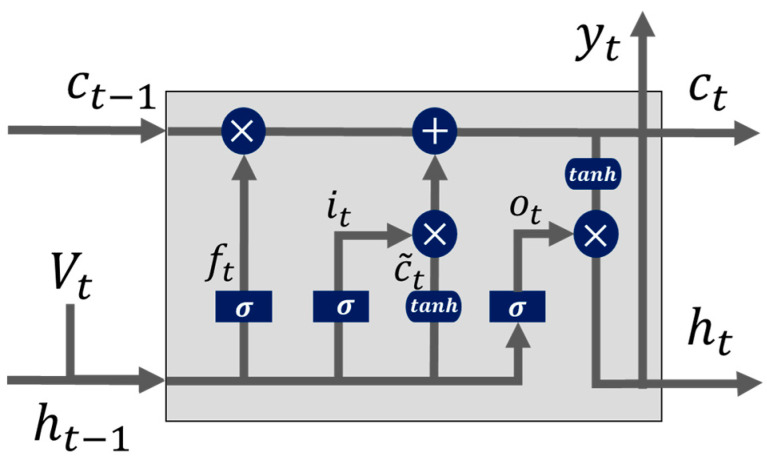
The structure of the LSTM model [34].

Long Short-Term Memory (LSTM). LSTM neural networks are a type of recurrent neural network (RNN) capable of learning order dependence in forecasting problems. LSTM has successively addressed the vanishing gradient problem of RNNs by introducing cell states [32,33]. We utilized LSTM as a univariate model to forecast ED patient arrivals. In Figure 2, the forward propagation of time-series data in LSTMs is illustrated.

Given an input time series $v=\left\{v_{1},\;v_{2},\ldots \;,\;v_{T}\right\}$, the LSTM network maps the input time-series data to two output time sequences, $h=\left\{h_{1},\;h_{2},\ldots \;,\;h_{T}\right\}$ and $y=\left\{y_{1},\;y_{2},\ldots \;,\;y_{T}\right\}$, iteratively by updating the states of memory cells with the following procedure. First, as shown in Figure 2, the forget gate is applied to help the LSTM network decide how to process information from the cell state. A sigmoid function σ(·) is applied to calculate the activation of the forget gate as [18]:

(17) $$f_{t}=\sigma \left(W_{fv}v_{t}+W_{fh}h_{t-1}+W_{fc}C_{t-1}+b_{f}\right)$$

The output, $f_{t}$, from Equation (17) is a value between 0 and 1, corresponding to the last cell state, $C_{t-1}$. The value 0 results in forgetting the last state completely, while the value 1 stands for keeping the last state completely. Next, the LSTM model decides the new information to be stored in the new cell state by utilizing a sigmoid layer. The input gate layer, $i_{t}$, is represented as

(18) $$i_{t}=\sigma \left(W_{iv}v_{t}+W_{ih}h_{t-1}+W_{ic}C_{t-1}+b_{i}\right)$$

The input gate identifies the information to be updated. The tanh function constructs a vector, ${\tilde{C}}_{t}$, to store the new values, which is added to the new cell state as

(19) $${\tilde{C}}_{t}=tanh\left(W_{cv}v_{t}+W_{ch}h_{t-1}+b_{c}\right)$$

The old cell state, $C_{t-1}$, is updated with the estimated $f_{t}$ and ${\tilde{C}}_{t}$ values. Specifically, the old cell state is multiplied with $f_{t}$ in order to forget information from the last state. The new values are multiplied with the input gate layer to decide how much new information should be updated to the new cell state, presented in Equation (20)

(20) $$C_{t}={\tilde{C}}_{t}\times i_{t}+C_{t-1}f_{t}$$

Another sigmoid layer, σ(·), is used as the output gate to filter and output the cell state as $o_{t}$, given as

(21) $$o_{t}=\sigma \left(W_{ov}v_{t}+W_{oh}h_{t-1}+W_{oc}C_{t-1}+b_{o}\right)$$

A cell output tanh activation function is also applied over the cell state and multiplied by the output, $o_{t}$, to give the desired result.

(22) $$h_{t}=o_{t}\times tanh\left(C_{t}\right)$$

Notations $W_{i},\;W_{f},\;W_{o},\;W_{c}$ and $b_{i},\;b_{f},b_{o},\;b_{c}$ represent the weights and biases associated with the input gate, forget gate, output gate, and cell state within Equations (17)–(21), respectively. $h_{t-1}$ is the hidden state output at time t−1, $v_{t}$ is the input at time t, and $C_{t}$ is the intermediate cell state of the network. For the multivariable LSTM model, additional variables such as the maximum temperature, average temperature, minimum temperature, pressure, humidity, and precipitation were utilized.

We divided the data into training (90%) and testing (10%) datasets to train and evaluate the forecasting models. As the training and validation aspects were not significantly affected by the forecasting horizons, the same approaches were applied for the training models based on the remaining forecasting horizons of 7, 14, 21, and 30 days.

### 2.4. Model Evaluation Criteria

The mean absolute error (MAE) and root mean squared error (RMSE) are frequently used to evaluate the performance of supervised learning algorithms by comparing predicted values against observations. MAE denotes the mean absolute difference between the predicted ED patient arrival and the observed values, whereas RMSE is the average root mean squared error between the predicted and observed values. Although RMSE and MAE are appropriate quality measures to assess the average model performance error, RMSE better penalizes larger discrepancies, whereas MAE provides easier interpretation. The goal of this study was to select a model that provides low RMSE and MAE values, as it shows that the given forecasting model is able to fit the time-series data. The developed algorithms were compared to select the best forecasting model with the smallest forecast error. All algorithms were implemented in Python (v. 3.8).

## 3. Results

### 3.1. Statistical Data Analysis

**Figure 3:**
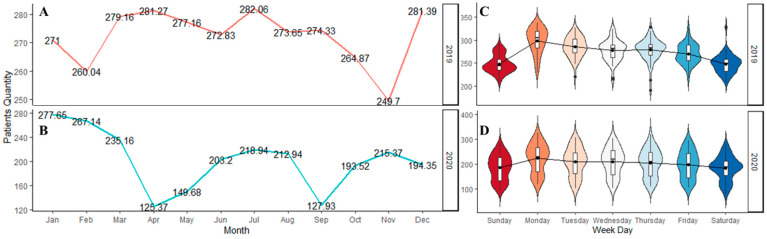
The ED patient arrivals for the two years. The figure depicts: (**A**) The average arrivals for 2019; (**B**) The average arrivals for 2020; (**C**) The weekday arrivals for 2019; and (**D**) The weekday arrivals for 2020.

**Figure 4:**
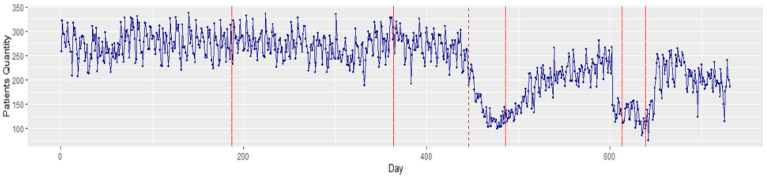
Time series plot of total ED patient visits for 2019–2020. The solid red lines depict the changepoints that occurred in the time series data, while the red dash line depicts the start of the COVID lockdown in Michigan, USA.

There were a total of 173,285 patient arrivals to the ED between 1 January 2019 and 31 December 2020. A total of 2191 patients tested positive for COVID-19 upon arrival to the ED. The mean age of patients arriving to the ED was 47.1 ± 18.8 years. A majority were female (88,679, 51.2%), and 123,721 (71.4%) were Black. The average ED arrival per day was 237.1 (SD 56.6) patients. Figure 3 illustrates the average ED patient arrival by month and weekdays. Overall, the high variation in monthly patient arrivals reflects the impact of the COVID-19 pandemic. Daily variation reflects the expected peak arrivals on Mondays and a nadir on weekends. Figure 4 shows a time series plot of the total daily ED visits over the two-year time frame.

**Figure 5:**
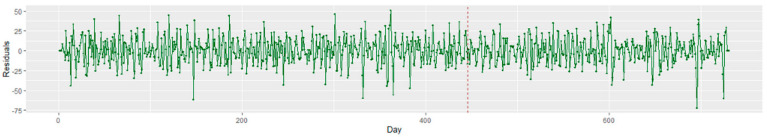
Time series plot of the stationary ED patient visits for 2019 to 2020. The red dash line depicts the start of the COVID lockdown in Michigan, USA.

Viewing these arrivals with a Bayesian changepoint analysis, significant changes were notable between December 2019 and January 2020. In addition, a major change was observed on day 446 (21 March 2020) owing to the strict COVID-19 lockdown measures instituted in Michigan. The existence of these changepoints illustrates the nonstationary behavior of the data. As stationarity has a tremendous influence on how the data is perceived and predicted, first-order differencing is applied to stabilize the time-series mean and/or variance. Figure 5 displays the transformed stationary data after first-order differencing.

The ADF results indicate that the dataset was stationary (*p* > 0.05). Thus, the differenced series presented a stationary trend: the mean, variance, and autocorrelation did not change significantly during the overall observation time. First-order differencing was only applied to the SARIMA and SARIMAX models. The HW, FP, and LSTM models do not require the time-series models to be differenced or stationary.

**Figure 6:**
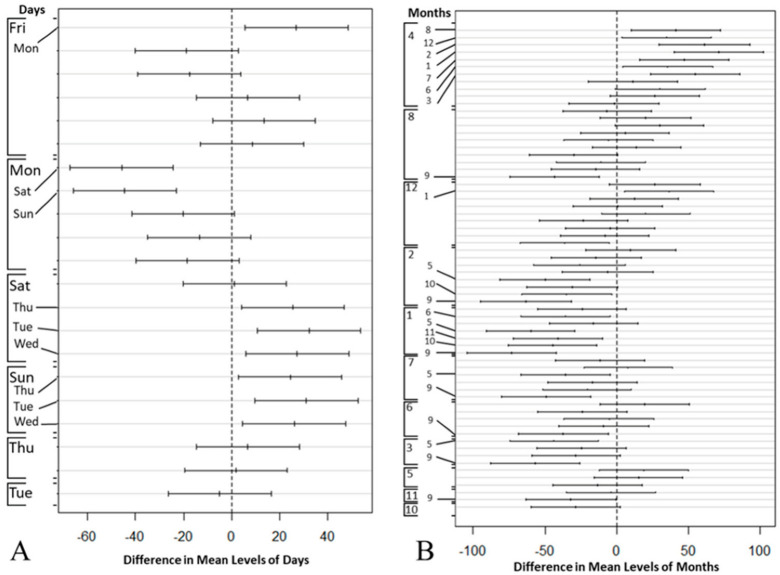
Tukey’s test: compare the mean difference of ED patient arrivals by (**A**) week day and (**B**) month.

The results of the Two-way ANOVA illustrate that although there were at least a weekday (*p* < 0.05) and a month (*p* < 0.05) that significantly impacted the patient’s arrival, no interaction could be detected between the weekday and the month factors. Figure 6 demonstrates Tukey’s HSD test results, including a total of 21 and 66 pairwise comparisons for weekdays and months in part (a) and (b), respectively. Both parts (a) and (b) plot the confidence interval for the difference in means between the pairs, and significantly different levels (i.e., *p* < 0.05) of weekdays and month are mentioned on the left margin of each plot. A statistically significant difference (*p* < 0.05) could be found between weekdays and weekends.

The Spearman correlation showed that only humidity (r = 0.11) had a positive correlation that was statistically significant (*p* < 0.05) with ED patient arrivals. Maximum temperature (r = −0.10), average temperature (r = −0.10), minimum temperature (r = −0.09), COVID lockdown (r = −0.77), and precipitation (r = −0.54) were statistically significant (*p* < 0.05) with a negative correlation to ED patient arrivals. We used these significant variables to develop the multivariable forecasting models.

### 3.2. Forecasting Models

#### 3.2.1. Univariate Models: SARIMA, FP, HW, and LSTM

Potential parameters (i.e., p, d, q values) for the SARIMA model were generated using the autocorrelation and the partial autocorrelation functions. An Auto ARIMA time series function was utilized to select an optimal order for the model by automatically iterating through different combinations of p, d, q parameters based on a grid search algorithm. The Auto ARIMA function returns the best SARIMA model according to the smallest Akaike information criterion (AIC) or Bayesian information criterion (BIC). The function searches for possible models within the order constraints provided.

The SARIMA with parameters (2,1,2)(1,0,[2])7 presented the smallest AIC value of 6606 (Table A1). The non-seasonal element gave a trend autoregression order, *p* = 2; a trend differencing order, d = 1, which calculates the first order non-seasonal differencing; and a trend moving average order, q = 2. The SARIMA model justified our ADF results as it showed that the time series data were not stationary; hence, a differencing of lag 1 was applied to achieve stationarity. The seasonal element gave a seasonal autoregressive order, *p* = 1, which made use of the first seasonally offset observation in the model; a D = 0, which indicates that the seasonality was stationary and that no seasonal differencing was required; and a Q = 2, which would use first-order errors in the model (e.g., moving average). The model gave the m value, the number of periods in a seasonal cycle, as 7 days.

Table A1 reports the estimated value of the coefficients of the model, their relative standard errors, and significance level. The intercept value was not significant and did not produce the average value of ED patient arrival during the forecast horizon. The non-seasonal and seasonal autoregressive orders were statistically significant (*p* < 0.05), but the first order non-seasonal moving average (ma.L1) was not statistically significant, so we proceeded to use the second-order non-seasonal moving average coefficient (ma.L2), which was statistically significant. The seasonal moving average (ma.S.L7 and ma.S.L14) coefficients were statistically significant. The parameter estimates ar.L1, ar.L2, ma.L2, ar.S.L7, and ma.S.L14 were the features that significantly impacted the time series data.

A non-exhaustive grid search was applied to achieve the best values for a univariate FP model’s parameter. The changepoint prior and seasonality prior scales were tuned, which determined the flexibility of the trend and seasonality. The model automatically captured the weekly seasonal trends based on the priors. The optimal values for the parameters consisted of a changepoint prior scale of 0.01 and a seasonality prior scale = 1.0.

The HW forecast model determined by the seasonal multiplicative HW method (i.e., refer to Equations (13)–(16)) gave the following smoothing parameters: α = 0.384, β = 4.94 × 10^−12^ and γ = 9.88 × 10^−12^, with the AIC value as low as 4135. The seasonality component, *st*, gave a value of 7, representing a weekly cycle for the time series. The alpha (α) value was similar to the moving average, which shows how the weights adjusted the amount of smoothing by defining how each component reacts to the current time series conditions. Lower smoothing weights give less weight to recent data and vice versa. Thus, adjusting the weight of the α component usually has the best chance of improving the accuracy measures.

**Figure A1:**
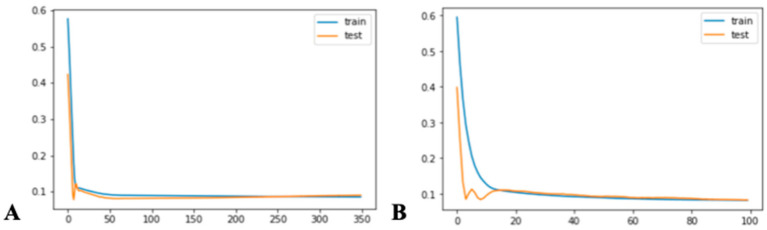
The loss function for ED patient arrival of the best LSTM: (**A**) univariate and (**B**) multivariate.

For the LSTM model, a grid search was employed to tune the model. The weights and biases in each gate were updated with the backpropagation algorithm. The model’s optimal parameters included an Adam optimizer, batch size of 70, hidden layer of 1, and 350 epochs. As shown in Figure A1a, the model was trained over 350 epochs to achieve stationary loss, leading to RMSE and MAE scores of 29.92 and 23.64, respectively.

**Figure 7:**
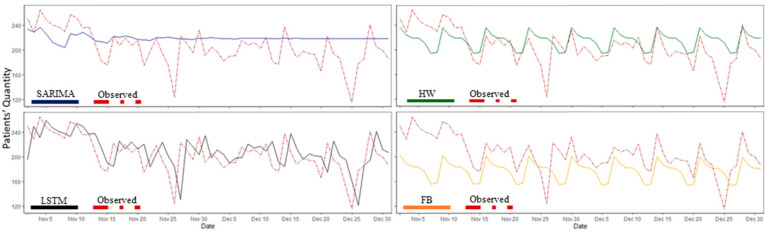
The univariate model predicted values vs. observed data (i.e., test data) for patient arrivals with a one-day forecast horizon. SARIMA: seasonal autoregressive integrated moving average, FP: Facebook Prophet, HW: Holt–Winters, and LSTM: Long Short-Term Memory.

Figure 7 shows a graphical comparison between the observed data (i.e., test data) and the forecasted SARIMA, FB, HW, and LSTM models using a 1-day horizon. Table 3 shows the RMSE and MAE scores for each model with varying performances in different forecasting horizons. The results illustrate that HW outperformed all other models in short-term predictions (1–7 days), LSTM performed best in long-term predictions (21 days or more), and SARIMA displayed the best performance in the forecasting horizon of 14 days. FP had a weak prediction compared with the other models in different forecast horizons.

Table 3 presents the results of the univariate models across different forecasting horizons. For example, across a seven-day forecasting horizon, the observed average ED daily arrivals were 207 patients, and the HW model estimated 216 (SD ± 28.19) patients (mean absolute percent error of 4.3%). The MAE values implied that, on average, the HW forecast error from the true daily patient arrival rate was 21.32.

#### 3.2.2. Multivariable Models: SARIMAX, FP, and LSTM

A SARIMAX model was fitted to ED patient arrival data, as shown in Table A2. The SARIMAX model parameters were tuned using a grid search. The best (p, d, q)(P, D, Q)m parameters provided an AIC score of 6200 for a SARIMAX model with parameters (2, 0, 1)(2, 0, [])7. The non-seasonal element for the SARIMAX model gave a trend autoregression order, *p* = 2; a trend differencing order, d = 0, which means no differencing; and a trend moving average order, q = 1. The seasonal element gave a seasonal autoregressive order, *p* = 2, which makes use of the second seasonally offset observation in the model, and D = 0, which indicates that the seasonality was stationary and that no seasonal differencing was required. The model gave the m value, the number of periods in a seasonal cycle, as 7 days.

Table A2 displays the estimated value of the model coefficients, the relative standard errors, and significance level. Lockdown and average temperature were the only significant variables that contributed to the model. The non-seasonal and seasonal autoregressive orders were statistically significant (*p* < 0.05). Likewise, the first order non-seasonal moving average (ma.L1) coefficient was statistically significant (*p* < 0.05). These parameter estimates have a significant impact on the time series data.

For the FP model, a non-exhaustive grid search was applied to tune the model. The optimal values for the parameters were as follows: changepoint prior scale = 0.05; seasonality prior scale = 10; and a weekly seasonal trend, resulting in RMSE and MAE scores of 48.68 and 43.25, respectively.

The exogenous LSTM was fine-tuned using a grid search approach. The model’s optimal parameters included an Adam optimizer, batch size of 72, a hidden layer of 1, and 50 epochs. Figure A1b demonstrates a decrease in loss function over 50 epochs. The exogenous LSTM model achieved RMSE and MAE scores of 28.55 and 20.52, respectively.

**Figure 8:**
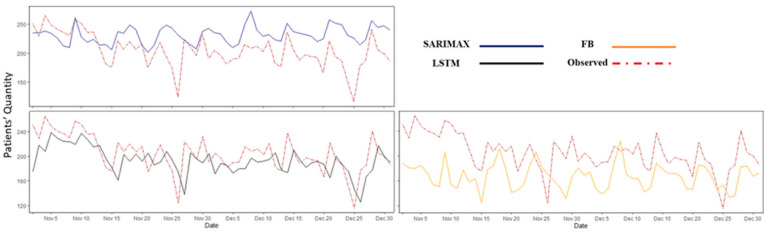
The multivariable model predicted values vs. observed data (i.e., test data) for the ED patient arrival with a one-day forecast horizon. SARIMAX: seasonal autoregressive integrated moving average exogenous, LSTM: Long Short-Term Memory, and FP: Facebook Prophet.

As shown in Figure 8, the multivariable models performed more accurately than the univariate models did. In Table 4, the values of the performance measures (i.e., RMSE and MAE) are reported for the multivariable forecasting models over different forecasting horizons. LSTM exhibited the best overall performance among the multivariable models. Forecast modeling with SARIMAX and FP did not perform well for the extended time horizons.

The interpretation of the results in Table 4 is analogous to that in Table 3. For a seven-day forecasting horizon, the observed average ED daily arrivals were 207 patients, and the exogenous LSTM model estimated 195 (SD ± 30.04) patients (mean absolute percent error of 5.8%). The MAE values imply that, on average, the exogenous LSTM forecast distance from the true daily patient arrival rate was 21.32. See Appendix A for detailed results of the forecasting models.

## 4. Discussion

The COVID-19 pandemic has exposed the healthcare system’s poor ability to predict surges in ED arrivals and match resources and staffing accordingly. This research addresses the problem of time-series modeling of ED patient arrival through extreme swings during the COVID-19 pandemic. Using univariate and multivariable forecasting methods, this study established a framework to improve future resource planning for EDs and hospitals.

The major contribution of this research is the development of forecasting models capable of quickly adjusting to unexpected changes in the trends of ED patient arrivals during a medical surge, such as that occurring during a pandemic. Previous time-series studies have established the existence of seasonal and weekly variations in ED patient arrival patterns prior to the pandemic [35,36,37]. Limited data addresses forecasting during a pandemic [38]. Our study shows that seasonal and weekly patterns of daily demand for ED services are maintained during the pandemic. Furthermore, time-series models can accurately forecast ED visits during short- and long-term forecast horizons. The forecasting accuracy depends on the specific model employed and the length of the time horizon.

Our feature selection analysis showed that only humidity was positively correlated and statistically significant with patient arrivals. Temperature, precipitation, and COVID lockdown were negatively correlated and statistically significant with patient arrivals. We hypothesized that incorporating additional climatic factors in the multivariable models would improve forecasting accuracy, as has been previously reported [21,39]. Furthermore, we observed the negative influence of COVID lockdown (i.e., disease outbreak) on patient arrivals, especially during the early days of the pandemic. Nevertheless, the univariate models performed best in this study.

Our forecasting results illustrate that univariate HW modeling performed well, with an average RMSE of 28.3 patients for short-term predictions (1–7 days), and LSTM modeling, which runs on recursive neural networks, performed best in long-term predictions (>21 days) with an average RMSE of 30.9 patients. The average MAE for these models was 21.3 to 24.5, indicating a 7–10% absolute error in forecasting arrivals, depending on the time horizon. Highly accurate short-term models may be most useful in situations where resources can be shifted relatively quickly, such as on-call staffing. Models that are more accurate over a longer horizon are likely to be useful for staff scheduling, supply readiness, and the preparation of additional treatment beds or areas.

This study has several limitations. First, we investigated data from a single hospital (i.e., urban academic ED) located in Michigan. With slight modifications to the forecasting models, it can be generalizable and scalable to other hospital settings and ED. Furthermore, the data contained ED patient arrivals before and during COVID-19, which may have influenced the forecasting efficacy of different methods. Second, our results can only be generalized to hospitals within our geographic regions because of the climatic characteristics used in the study. Future studies will extend this model to regions with different climatic characteristics. Third, this study did not consider the impact of holidays and ED diversion status during the pandemic, and their inclusion may improve the model fit. Lastly, the study did not assess the classification of patients according to diagnosis or severity. In future studies, we will utilize the vector autoregressive moving average to forecast multiple time-series models of ED arrivals based on the severity of illness and diagnoses.

## 5. Conclusions

Forecasting models are promising tools for predicting trends in ED patient arrivals during significant swings caused by the pandemic. Further model validation across diverse populations and time horizons may create a framework for improved resource matching to forecasted patient arrivals.

## Figures and Tables

**Table 1:** Benefits and limitations of selected forecasting models.

Models	Benefits	Limitations
SARIMA/ SARIMAX	Solid mathematical and statistical theory. Time-varying trends/seasonal patterns. Relatively few parameters. Handles exogenous variables.	Difficulty tuning the model parameters. Usually computationally expensive. Prone to overfitting.
FP	Supports seasonality with multiple periods. Robust to missing data. Does not require data interpolation. Handles outliers. Handles exogenous variables.	Does not consider multiplicative models. Strict formatting requirement Restricted to Gaussian noise distribution. Does not take autocorrelation into account. Does not assume a stochastic trend.
HW	Works best for data with trends and with seasonality that increases over time. The results are interpretable. Very easy to implement.	The presence of outliers distorts the results. Not expanded to multivariable approach. Accounts for only a single seasonal pattern.
LSTM	Learns information for an extended period. Mitigates the vanishing gradient problem. No specific assumptions. Handles exogenous variables.	Computationally time-consuming. Sensitive to random weight initializations. Prone to overfitting.

SARIMA: seasonal autoregressive integrated moving average; FP: Facebook Prophet; HW: Holt-Winters; LSTM: Long Short-Term Memory; and SARIMAX: seasonal autoregressive integrated moving average exogenous.

**Table 2:** Exogenous variables.

Factors	Variables	Explanation
Disease Outbreak	COVID lockdown	Denoting whether the COVID lockdown was in place or not in Michigan
Climatic	Average temperature	The average temperature (K)
	Minimum temperature	The minimum temperature (K)
	Maximum temperature	The maximum temperature (K)
	Precipitation	Quantity of water deposited (i.e., rain, snow, or hail)
	Relative humidity	Percentage of relative humidity
	Pressure	Pressure within the earth atmosphere (Hg)

Note: K—Kelvin, Hg—Barometric pressure.

**Table 3:** RMSE and MAE values for univariate models in five different forecasting horizons.

Models	Forecasting Horizon (in Days)									
	1		7		14		21		30	
	RMSE	MAE	RMSE	MAE	RMSE	MAE	RMSE	MAE	RMSE	MAE
SARIMA	33.57	26.58	32.73	26.03	28.81	22.28	47.59	39.97	96.20	89.92
FP	43.82	34.74	45.94	41.62	54.50	51.15	60.67	57.27	53.75	49.99
HW	28.42	21.29	28.19	21.32	30.20	23.07	38.47	32.34	89.74	84.09
LSTM	29.92	23.64	29.94	23.65	30.70	23.92	30.43	23.97	31.32	24.52

Note: Green highlights the model with the best results. Interpretation: The LTSM model with a 30-day horizon had the lowest RMSE of 31.32 and lowest MAE of 24.52 of all four models, suggesting that the prediction of daily patients on a 30-day horizon was best accomplished with the LTSM model. MAE, mean absolute error; RMSE, root mean square error (units = patients).

**Table 4:** RMSE and MAE values for multivariable models in five different forecasting horizons.

Models	Forecasting Horizon (in days)									
	1		7		14		21		30	
	RMSE	MAE	RMSE	MAE	RMSE	MAE	RMSE	MAE	RMSE	MAE
SARIMAX	35.57	31.08	39.76	34.75	48.27	43.02	52.89	46.96	60.92	53.91
FP	48.68	43.25	58.27	53.37	70.07	65.56	80.34	76.13	78.00	72.39
LSTM	28.55	20.52	30.04	21.32	31.26	22.14	31.20	23.54	35.96	28.03

Note: Green highlights the model with the best results. Interpretation: The LSTM model had the smallest RMSE and MAE values for the 30-day forecasting horizon. MAE, mean absolute error; RMSE, root mean square error (units = patients).

## Data Availability

Not applicable.

## Appendix A. Forecasting Modeling Results

**Table A1:** Parameter Estimates for SARIMA (2,1,2)(1,0,[2])7 with a One Day Forecast Time Horizon.

Parameter	Estimated Value	Standard Error	*p*-Value
Intercept	0.000	0.002	0.940
ar.L1	−0.644	0.226	0.004 *
ar.L2	0.216	0.056	0.000 *
ma.L1	0.095	0.225	0.672
ma.L2	−0.662	0.159	0.000 *
ar.S.L7	0.999	0.001	0.000 *
ma.S.L7	−0.857	0.039	0.000 *
ma.S.L14	−0.114	0.040	0.004 *

* significant at *p* < 0.05.

**Table A2:** Parameter estimates for SARIMAX (2, 0, 1)(1, 0, [])7 with a one day forecast horizon.

Parameter	Estimated Value	Standard Error	*p*-Value
Lockdown	−104.845	8.061	0.000 *
Humidity	0.078	0.089	0.382
Max. Temp	−0.761	0.570	0.182
Avg. Temp	2.131	0.930	0.022 *
Min. Temp	−0.421	0.506	0.405
Precipitation	0.174	0.311	0.577
ar.L1	1.490	0.035	0.000 *
ar.L2	−0.493	0.031	0.000 *
ma.L1	−0.999	0.280	0.000 *
ar.S.L7	0.418	0.039	0.000 *
ar.S.L14	0.173	0.040	0.000 *

* significant at *p* < 0.05.

## References

[1] Woodruff A., Frakt A.B. (2020). COVID-19 Pandemic Leads to Decrease in Emergency Department Wait Times. Proc. JAMA Health Forum.

[2] Dugas A.F., Morton M., Beard R., Pines J.M., Bayram J.D., Hsieh Y.-H., Kelen G., Uscher-Pines L., Jeng K., Cole G. (2013). Interventions to mitigate emergency department and hospital crowding during an infectious respiratory disease outbreak: Results from an expert panel. PLoS Curr..

[3] Sullivan C., Staib A., Khanna S., Good N.M., Boyle J., Cattell R., Heiniger L., Griffin B.R., Bell A.J., Lind J. (2016). The National Emergency Access Target (NEAT) and the 4-hour rule: Time to review the target. Med. J. Aust..

[4] Carr B.G., Kaye A.J., Wiebe D.J., Gracias V.H., Schwab C.W., Reilly P.M. (2007). Emergency department length of stay: A major risk factor for pneumonia in intubated blunt trauma patients. J. Trauma Acute Care Surg..

[5] Robertson J.J., Long B. (2018). Suffering in silence: Medical error and its impact on health care providers. J. Emerg. Med..

[6] Hall L.H., Johnson J., Watt I., Tsipa A., O’Connor D.B. (2016). Healthcare staff wellbeing, burnout, and patient safety: A systematic review. PLoS ONE.

[7] Gul M., Celik E. (2020). An exhaustive review and analysis on applications of statistical forecasting in hospital emergency departments. Health Syst..

[8] Marcilio I., Hajat S., Gouveia N. (2013). Forecasting daily emergency department visits using calendar variables and ambient temperature readings. Acad. Emerg. Med..

[9] Batal H., Tench J., McMillan S., Adams J., Mehler P.S. (2001). Predicting patient visits to an urgent care clinic using calendar variables. Acad. Emerg. Med..

[10] Giannakeas V., Bhatia D., Warkentin M.T., Bogoch I., Stall N.M. (2020). Estimating the maximum daily number of incident COVID-19 cases manageable by a healthcare system. MedRxiv.

[11] Zhang Y., Zhang J., Tao M., Shu J., Zhu D. (2022). Forecasting patient arrivals at emergency department using calendar and meteorological information. Appl. Intell..

[12] Corcuera Hotz I., Hajat S. (2020). The effects of temperature on accident and emergency department attendances in London: A time-series regression analysis. Int. J. Environ. Res. Public Health.

[13] Chan E.Y., Goggins W.B., Yue J.S., Lee P. (2013). Hospital admissions as a function of temperature, other weather phenomena and pollution levels in an urban setting in China. Bull. World Health Organ..

[14] Linares C., Diaz J. (2008). Impact of high temperatures on hospital admissions: Comparative analysis with previous studies about mortality (Madrid). Eur. J. Public Health.

[15] Wargon M., Casalino E., Guidet B. (2010). From model to forecasting: A multicenter study in emergency departments. Acad. Emerg. Med..

[16] Sato R.C. (2013). Disease management with ARIMA model in time series. Einstein.

[17] Qiu M., Song Y. (2016). Predicting the direction of stock market index movement using an optimized artificial neural network model. PLoS ONE.

[18] Toharudin T., Pontoh R.S., Caraka R.E., Zahroh S., Lee Y., Chen R.C. (2020). Employing long short-term memory and Facebook prophet model in air temperature forecasting. Commun. Stat.-Simul. Comput..

[19] Zhang X., Pang Y., Cui M., Stallones L., Xiang H. (2015). Forecasting mortality of road traffic injuries in China using seasonal autoregressive integrated moving average model. Ann. Epidemiol..

[20] Service N.W. NOWDATA—NOAA Online Weather Data. https://www.weather.gov/wrh/Climate?wfo=dtx.

[21] Calegari R., Fogliatto F.S., Lucini F.R., Neyeloff J., Kuchenbecker R.S., Schaan B.D. (2016). Forecasting daily volume and acuity of patients in the emergency department. Comput. Math. Methods Med..

[22] Jones S.S., Thomas A., Evans R.S., Welch S.J., Haug P.J., Snow G.L. (2008). Forecasting daily patient volumes in the emergency department. Acad. Emerg. Med..

[23] Romero T. Americans Fear Hospital Visits Amid the COVID-19 Crisis. The Beach? Not So Much. Philly Voice Newspaper.

[24] Michigan.gov Coronavirus—Michigan Data. https://www.michigan.gov/coronavirus/0,9753,7-406-98163_98173---,00.html.

[25] Zhang X., Liu Y., Yang M., Zhang T., Young A.A., Li X. (2013). Comparative study of four time series methods in forecasting typhoid fever incidence in China. PLoS ONE.

[26] Kam H.J., Sung J.O., Park R.W. (2010). Prediction of daily patient numbers for a regional emergency medical center using time series analysis. Healthc. Inform. Res..

[27] Arunraj N.S., Ahrens D., Fernandes M. (2016). Application of SARIMAX model to forecast daily sales in food retail industry. Int. J. Oper. Res. Inf. Syst..

[28] Cools M., Moons E., Wets G. (2009). Investigating the variability in daily traffic counts through use of ARIMAX and SARIMAX models: Assessing the effect of holidays on two site locations. Transp. Res. Rec..

[29] Taylor S.J., Letham B. (2018). Forecasting at scale. Am. Stat..

[30] Champion R., Kinsman L.D., Lee G.A., Masman K.A., May E.A., Mills T.M., Taylor M.D., Thomas P.R., Williams R.J. (2007). Forecasting emergency department presentations. Aust. Health Rev..

[31] Koehler A.B., Snyder R.D., Ord J.K. (2001). Forecasting models and prediction intervals for the multiplicative Holt–Winters method. Int. J. Forecast..

[32] Le X.-H., Ho H.V., Lee G., Jung S. (2019). Application of long short-term memory (LSTM) neural network for flood forecasting. Water.

[33] Zheng J., Xu C., Zhang Z., Li X. Electric load forecasting in smart grids using long-short-term-memory based recurrent neural network. Proceedings of the 2017 51st Annual Conference on Information Sciences and Systems (CISS).

[34] Masoud S., Mariscal N., Huang Y., Zhu M. (2021). A Sensor-Based Data Driven Framework to Investigate PM 2.5 in the Greater Detroit Area. IEEE Sens. J..

[35] Upshur R.E., Moineddin R., Crighton E., Kiefer L., Mamdani M. (2005). Simplicity within complexity: Seasonality and predictability of hospital admissions in the province of Ontario 1988–2001, a population-based analysis. BMC Health Serv. Res..

[36] Jones S.A., Joy M.P., Pearson J. (2002). Forecasting demand of emergency care. Health Care Manag. Sci..

[37] Downing A., Wilson R. (2002). Temporal and demographic variations in attendance at accident and emergency departments. Emerg. Med. J..

[38] Duarte D., Walshaw C., Ramesh N. (2021). A comparison of time-series predictions for healthcare emergency department indicators and the impact of COVID-19. Appl. Sci..

[39] Wargon M., Guidet B., Hoang T., Hejblum G. (2009). A systematic review of models for forecasting the number of emergency department visits. Emerg. Med. J..

